# Novel Approaches to Program Cells to Differentiate into Cardiomyocytes in Myocardial Regeneration

**DOI:** 10.31083/j.rcm2312392

**Published:** 2022-11-30

**Authors:** Victor Bonavida, Kaitlyn Ghassemi, Gwendolyn Ung, Keiko Inouye, Finosh G Thankam, Devendra K Agrawal

**Affiliations:** ^1^Department of Translational Research, Western University of Health Sciences, Pomona, CA 91766, USA

**Keywords:** heart failure, cardiac regeneration, cell programming, iPSC, cell technologies

## Abstract

With heart failure (HF) being one of the leading causes of hospitalization and death worldwide, multiple stem cell therapies have been attempted to accelerate the regeneration of the infarct zone. Versatile strategies have emerged to establish the cell candidates of cardiomyocyte lineage for regenerative cardiology. This article illustrates critical insights into the emerging technologies, current approaches, and translational promises on the programming of diverse cell types for cardiac regeneration.

## 1. Introduction

More than 6.2 million adult Americans beyond 20 years of age are suffering from heart failure (HF) [1] thus significantly contributing to the global burden of 38 million patients worldwide [2]. While there have been substantial improvements in pharmacological and clinical interventions to treat HF, half of the patient population suffering from HF surrender to death within 5 years of diagnosis [3]. Moreover, roughly $30.7 billion have been spent annually on the healthcare services and treatment of HF and to meet the patients’ healthcare needs [3]. Fundamentally, regeneration and repair of the myocardium following ischemic events emerge as crucial areas of medical research. Motivated by the observation that the aging population is bound to acquire new cardiac-related diseases, multiple therapies for cardiovascular regenerative medicine are being explored on the otherwise terminally differentiated cardiac tissue. The human cardiomyocytes (CM) have a progressively slow turnover rate [4] with the most active events occurring in the first decade of life [2]. Therefore, the impetus for research into CM regeneration is clear, which relies on the understanding of the cells utilized in cardiac repair.

Exploring and altering the mechanisms of diverse cells, such as fibroblasts, endothelial cells, satellite cells, and stem cells, serve as valuable methods to gain insight in developing better therapeutics for cardiac tissue healing. Importantly, the cell programming strategies utilizing chemical and genetic material, cell structure, and proteins paved the way to versatile differentiation strategies [2]. The current understanding regarding the cell mechanisms to induce cell alterations highlights the significant progress in regenerative cardiology. Moreover, cell reprogramming to modify the cell physiology and functions through gene manipulations such as CRISPR, RNA interference, forced transcription factors, and Knockout/in have unveiled translationally worthwhile outcomes. This article brings insights into the versatile reprogramming approaches on diverse cell types for cardiac regeneration.

## 2. Cells and Mediators in Cardiac Repair

Regeneration of cardiac tissue following an injury or death is a complex process involving multiple cell types and lineages, including, but not limited to, cardiomyocytes, fibroblasts, endothelial cells, hematopoietic cells, and satellite cells. The most crucial cell types are the beating elements, cardiomyocytes (CM), that function over multiple stimuli, exhibit complicated cell cycles, and induce sufficient contractile substrates following the ischemic injury depending on the developmental stage (embryonic, neonatal, or adult). The embryonic ischemic lesions are regenerative, whereas the lesions in neonatal tissue are hyperplastic leading to incomplete regeneration of the original structure as well as compensatory growth [5]. However, lesions in the adult heart have neither been restored nor replaced [5]. CM constitute 90% of the mature adult heart cell mass accounting for 40% of the proportion of total cells followed by fibroblasts, endothelial cells, and vascular smooth muscle cells. Non-CM cells retain the ability to divide in response to ischemic or mechanical stress forming popular targets for cardiac regeneration [6].

Arguably one of the multiphase cardiac regenerative processes occurs following myocardial infarction (MI) in which the damaged CM are replaced by fibrotic tissue contributed by proliferative fibroblasts. While the initial function of these fibroblasts is to prevent rupture of ventricular muscle, the extension of this damage into perivascular and interstitial spaces leads to fibrous scars causing detrimental effects on cardiac contractility and interferes with the normal electrical conduction of the heart, leading to re-entrant arrhythmias [7].

Stem cell cardiogenesis of adult CM with regenerative potential has been correlated with the gene expression profiles of early fetal cell progenitors, illustrating that development and regeneration exhibit similar biological events, bringing translational opportunities. Even though the ability of the heart to replace damaged myocardium with healthy new cells is limited, experimental evidence revealed that reactivating cell division or inhibiting cell death among populations of CM accelerate the survival responses [8]. Multipotent stem cell progenitors occurring in specialized regions of the heart, *ex vivo* cells, and circulating stem cells with cardiac differentiating potency have been attempted for relocation to the damaged heart muscle; however, lack of sufficient cardiac stem cells and ability to accurately pinpoint effective CM are challenging. As CM are terminally differentiated, their regenerative capacity is inadequate to restore lost myocardium [8, 9]. Also, the formation of fibrotic scar tissue is the primary protective mechanism against further injury [9]. Generally, the cardiac cell therapy involves introduction of new CM precursors: adult cardiac stem cells, mesenchymal stem cells, embryonically derived cells, and induced pluripotent-derived cells. Additionally, CM proliferation mediated by cell cycle mediator factors such as cyclin A2, cyclin dependent kinase inhibitors, and microRNAs (miRs-29, -30, -141, -195, -199a-3p, -590-3p) resulted in improved cardiac function [6].

Endothelial cells are crucial for maintaining vascular homeostasis and are involved in secreting factors such as growth factors, cytokines, and chemokines to attract endothelial progenitor cells (EPCs). Vascular endothelial growth factor (VEGF) and stromal derived factor-1 (SDF-1) interact with their respective receptors to promote EPC mobilization whereas tumor necrosis factor alpha (TNFα) accelerates EPC migration and incorporation into local vascular structures [10]. Also, platelet-derived growth factor receptor (PDGFR)-β promotes migration and induction of angiogenic properties of EPCs. Additionally, both nitric oxide (NO) and erythropoietin (EPO) contribute to mobilization of EPCs, whereas angiopoietin-1 (Ang-1) inhibits mobilization [10].

Satellite cells are the regenerative cells that provide nuclear material for developing myocytes. Interferon gamma (IFN-γ), a cytokine secreted by macrophages and CD8+ T cells and is critical for proper functioning of both innate and adaptive immunity, is involved in the signaling of satellite cells [11, 12]. Skeletal muscle with declining IFN-γ shows dysfunctions in satellite cells, and aged skeletal muscle tissue shows downregulation of the IFN-γ pathway. Reduced regeneration potential leads to increased muscle fibrosis following the injury, increased accumulation of abnormal extracellular matrix (ECM) deposition, and reduced functional outcomes of the myocardium [12, 13].

A study showed that fibroblasts are transdifferentiated into skeletal muscle *in vitro* and *in vivo* by overexpressing MyoD, a myogenic transcription factor [14]. The reprogramming of fibroblast cells into cardiomyocytes has been targeted as a potential approach for cardiac repair following the injury. Cardiac and dermal fibroblasts were induced with the cocktail of transcription factors, Gata4, Mef2c, and Tbx5, resulting in the trans-differentiation towards cardiomyocyte-like cells [15, 16]. Pro-fibrotic factors that are secreted by both damaged and adjacent myocardial cells increase secretion of local pro-fibrotic mediators such as TGF-β, which induces myofibroblast differentiation and increases ECM synthesis, supporting regeneration [7].

CD34+ hematopoietic stem cells (HSCs), endothelial progenitor cells (EPCs), and mesenchymal stem cells (MSCs) [17] augment the repair after ischemic injury in cardiac tissue by stimulating angiogenesis and reducing infarct expansion through remodeling and fibrosis [17]. Moreover, certain molecular pathways have been shown to be involved in cardiac remodeling and regeneration. Exercise has been well-established to exert cardiovascular benefits, which extend to post-injury states as well. Activation of the tyrosine kinases ErbB2 and ErbB4 by neuregulin-1, a signaling molecule in the EGF family, in response to exercise has been shown to activate PI3K/Akt signaling cascades that protect ventricular myocytes from apoptosis as evident from animal models [18]. Moreover, exercise increases circulating levels of catecholamines and subsequently the levels of β-adrenergic receptors including β3. These receptors then stimulate the phosphorylation of endothelial nitric oxide synthase and increase levels of NO metabolites such as nitrite and nitroso-thiols, exhibiting cardioprotective effects in ischemic hearts [18].

MicroRNAs (miRNAs) are emerging targets for therapeutic and diagnostic tools in the field of cardiovascular disease due to their ability to be introduced and differentially expressed in certain diseased tissue and alter disease course. Interestingly, the miRNA cluster miR-17-92 is involved with the cell cycle, apoptosis, and cell proliferation, and individual molecules have been shown to be involved in cardiac remodeling, proliferation, and growth, whereas their attenuation is associated with worse outcomes in various cardiomyopathies [18, 19]. The miR-15 family promotes myocyte proliferation and enhances cardiac function post-myocardial infarction in adult cardiac tissue [20]. Different families and clusters of miRNAs are involved with induction of pro-apoptotic proteins, heat shock proteins, and even genes responsible for synthesis of structural elements such as collagen and ECM proteins fibrillin and elastin. Working together in complex patterns, miRNAs influence regeneration after ischemic cardiac injury and form potential therapeutic targets [20].

Versatile miRNA molecules have been identified in CM differentiation. For instance, miR-1 and miR-133 function together for mesodermal formation and simultaneously inhibiting growth of non-muscle tissue during development. The miR-199a-3p, miR214-3p and miR-483-3p are expressed in mesodermal cell lines, suggesting that they are crucial for the differentiation of human ESCs to mesodermal cells. Importantly, subtype of miRNAs has been found to play a role in host gene function within the heart and are named myomiRNAs which include miR-208/Myh7 expressed within the embryonic heart while miR208a/Myh7 and miR-499-Myh7b are expressed within the adult heart [21, 22]. Lee *et al*. [23], demonstrated that the delivery of microRNAs, miR-125b-5p, miR-199a-5p, miR-221, and miR-222 in combination with human embryonic-stem-cell-derived cells to the infarcted heart improved growth, development, sarcomere length, negative membrane potential capacity and calcium tolerance, and increased cardiac muscle cell markers [22, 23]. Sluijter *et al*. [24] reported that cardiomyocyte progenitor cells (CMPC) enhanced their differentiation in the presence and modulation by miR-1 and miR-499 which coincided with the regulation of CMPC function, proliferation, and differentiation [21, 22, 24]. The key cells, cytokines, gene factors, and signaling molecules that are critically involved in cardiac repair are summarized in Table 1 (Ref. [7, 10, 11, 12, 14, 15, 16, 17, 18, 19, 21, 22, 24]).

**Table 1.** **Summary of many of the key cells, cytokines, factors, and molecules that play roles in cardiac repair**.

Cells	Target	Function	Reference
	Endothelial cell	Secretes factors to promote mobilization and function of EPCs	[10]
	Satellite cell	Provide nuclear material for developing myocytes	[11]
	Fibroblast	Potential to trans-differentiate into myocytes	[14, 15, 16]
	CD34+ hematopoietic stem cell	Stimulation of angiogenesis, remodeling, and fibrosis	[17]
	Endothelial progenitor cell	Stimulation of angiogenesis, remodeling, and fibrosis	[17]
	Mesenchymal stem cell	Stimulation of angiogenesis, remodeling, and fibrosis	[17]
Cytokines	Target	Function	Reference
	VEGF	Promotes EPC mobilization	[10]
	SDF-1	Promotes EPC mobilization	[10]
	PDGF-β	Promotes migration and induction of angiopoietic properties	[10]
	TNF-α	Accelerates EPC migration and incorporation into local vascular structures	[10]
	EPO	Promotes EPC mobilizations	[10]
	Ang-1	Inhibits EPC mobilization	[10]
	IFN-γ	Involved signaling of satellite cells; Downregulation associated with satellite cell dysfunction	[11, 12]
	TGF-β	Induces myofibroblast differentiation and increases ECM synthesis	[7]
Gene Factors	Target	Function	Reference
	MyoD	Trans-differentiation of fibroblasts into skeletal muscle	[14]
	miR-17-92	Involved in cell cycle, apoptosis, and cell proliferation	[18, 19]
	miR-133	Involved in mesodermal formation and inhibition of non-muscle tissue	[21, 22]
	miR-199a-3p	Potentially involved in differentiation of ESCs to mesodermal cells	[21, 22]
	miR-214-3p	Potentially involved in differentiation of ESCs to mesodermal cells	[21, 22]
	miR-483-3p	Potentially involved in differentiation of ESCs to mesodermal cells	[21, 22]
	miR-208/Myh7	Involved in gene function within the embryonic heart	[21, 22]
	miR-208a/Myh7	Involved in gene function within the adult heart	[21, 22]
	miR-499-Myh7b	Involved in gene function within the adult heart	[21, 22]
	miR-1	Involved in cardiomyocyte progenitor cell function, proliferation, and differentiation	[21, 22]
	miR-499	Involved in cardiomyocyte progenitor cell function, proliferation, and differentiation	[21, 22, 24]
Signaling Molecules	Target	Function	Reference
	Neuregulin-1	Binds to ErbB2 and ErbB4 and activates PI3K/Akt signaling to protect from apoptosis	[18]
	NO	Promote mobilization of EPCs	[10]
	NO metabolites	Exhibit cardioprotective effects	[18, 19]

## 3. Current Cardiac Cell Therapies

Bone marrow-derived mesenchymal stem cells: Existing cell therapies for regeneration of damaged cardiac tissues have already been documented to show promising results. The MSC-HF trial is a large, double-blind placebo-controlled trial that introduced autologous intramyocardial injections of bone marrow-derived mesenchymal stromal cells (MSC) in patients with ischemic heart failure. Improvement in the left ventricular end-systolic volume (LVESV) and reduction of myocardial scar tissue were predominant after 1 year. Additionally, the 4-year follow-up revealed significantly fewer hospitalizations in the MSC group compared to the placebo group, suggesting that the autologous intramyocardial injections of MSCs greatly improved myocardial function in chronic ischemic heart failure patients [25]. Importantly, the bone marrow-derived progenitor cells differentiate into multiple different cell types in the heart such as cardiac muscle cells as well as vascular cells, promoting blood flow and regeneration following the ischemia [26]. MSCs elicit paracrine signaling pathways, such as chemokines and cytokines, as well as cell adhesion molecules that activate signal transduction pathways accelerating cardiac regeneration [27]. For instance, MSCs overexpressing the survival gene *Akt1* (*Akt+* MSCs) are superior in eliciting regenerative responses in damaged myocardium, specifically to prevent ventricular remodeling and restoring cardiac function [28].

Cardiac stem progenitor cells (CSC): Endogenous cardiac stem cells (eCSCs) are a group of resident-specific cardiac progenitor cells that have defined and identifiable membrane markers. Even though the regenerative potential and myogenic role of CSCs in adult myocardium is in debate, the CSCs are potent myogenic precursors with potential to re-muscularize and revascularize cardiac tissue *in vivo*. *In vitro* and *in vivo* experiments have shown that these eCSCs possess the properties of tissue-specific stem cells including self-renewal, multipotentency, and clonogenicity. CSCs differentiate into the myocardial cell lineages such as cardiomyocytes, vascular smooth muscle cells, and endothelial cells [29]. In post-MI patients, aerobic exercise reduces or reverses the maladaptive cardiac remodeling due to exercise-induced increases in nitric oxide (NO) that promotes vasodilation and subsequent reduction in blood pressure. Exposure of exogenous NO to isolated mouse hearts showed a dose-dependent increase in cardiomyocyte structural proteins as well as the transient expression of cardiac-specific transcription factors (GATA-4 and Nkx2.5) with a concomitant upregulation of cardiac structural genes (*TnnT2, Myh7, Myh6*). In addition, an isolated monoculture of CSCs treated with exogenous NO showed significant reduction in Wnt/β-catenin driving the differentiation of CSCs towards cardiomyocyte lineage [29]. In addition, the statins, (Rosuvastatin, Simvastatin and Pravastatin) inhibit 3-hydroxy-3-methylglutaryl coenzyme A (HMG-CoA) reductase and increase clonal expansion of CSCs through Akt phosphorylation [30]. For instance, the Rosuvastatin treatment in rat-MI model displayed a significant increase in endogenous CSCs at the borders of the infarcted tissue compared with untreated controls. In addition, commitment of CSCs into the myocyte lineage by c-kit- and GATA-4-mediated signaling was prevalent suggesting the beneficial effects of CSC in human cardiovascular disease [30].

Epicardial progenitor cells (EPC): Epicardial cells are the mesothelial cells present in the most superficial layer of cells in the heart functioning in the formation of the embryonic heart by activating the progenitor cells that undergo epithelial-to-mesenchymal transition prior to their terminal differentiation towards nonmyocyte cell lineages. EPCs migrate into the subjacent myocardium leading to the development of coronary smooth muscle, coronary endothelium, pericytes, and cardiac fibroblasts. Hence, the myocardium and epicardium engage in paracrine and contact-dependent cell interactions for the growth and development of different heart compartments [31]. Eventually, the epicardium becomes dormant in an adult heart; however, the cardiac injury reactivates signaling cascades that stimulate the epithelial-to-mesenchymal transitions as seen in embryogenesis [32].

## 4. Cell Programming 

Cell differentiation is a tightly controlled mechanism. Interestingly, it has been found that the overexpression of MYOD, a normally expressed skeletal muscle transcription factor, converted embryonic fibroblasts to myoblasts in mice [33]. The process of converting cells from one lineage to another by utilizing genetic components to introduce new cellular functions to the original cell is called cell programming [34, 35]. The rapid advances in technologies to manipulate DNA and other biological molecules led to the emerging field of synthetic biology [34]. Many examples of cell programming exist in the literature, spanning from the potential of striated muscle from invertebrate jellyfish to newt eye lenses forming iris epithelial cells to adult embryonic stem cells possessing the ability to differentiate into other embryological germ layers [36]. The potential for cell programming in vertebrates, especially humans, in nonpathological conditions is much limited [36].

Within the realm of cell programming, there are various methods to change the fate of cells, including induced pluripotent stem cells (iPSC) and direct reprogramming. These two methods offer the unique ability to regenerate cardiomyocytes. Fortunately, iPSCs evade the ethical controversies that utilize human embryos [37] while giving a somatic cell the ability to become a transitional multi or pluripotent state forming any cell type depending on factors and mediators [35]. This indirect reprogramming generates target cells on a large scale and *ex vivo* production [35]. Direct reprogramming, however, proves to be a more efficient process for tissue repair, eliminating the need to be transformed into an intermediate state [35] while retaining the epigenetic hallmarks of the original cell, making this technique a suitable method for mimicking age-related disease [35]. The common cell programming methods are discussed in the following sections.

## 5. Induced Pluripotency

The understanding that differentiated cells retain their uniform genetic information from their early embryonic state led to the emergence of pluripotent cells. Moreover, the discovery that the transcription factors acting as essential regulators of mature cell type switch has contributed to induced pluripotency [38]. Generation of induced pluripotent stem cells (iPSC) allowed to explore the mechanisms of degenerative disorders including HF. Patient-specific iPSC-derived cardiomyocytes (iPSC-CMs) have been developed for personalized medicine, allowing a precise understanding of the patient-specific disease, and creating “patient in a dish” phenotype [39, 40]. Generally, the iPSCs have been generated from somatic cells derived from patients’ adipocytes, keratinocytes, peripheral or cord blood, amniotic tissues, and/or lipoaspirate [40, 41]. With the ectopic transfer of pluripotent transgenes, such as *Oct4, Sox2, KLF4, Nanog*, and *c-Myc*, these cells begin to resemble and mimic embryonic stem cells (ESCs), possessing the ability to divide indefinitely and become pluripotent [40, 41]. Additionally, CM have been generated with the treatment of nicotinamide to ESCs inducing the specification of cardiac mesoderm [42]. In a seminal study, the combination of ascorbic acid, glycogen synthase kinase 3 inhibitor CHIR99021, and bone morphogenetic protein 4 (BMP4) was used to convert iPSCs into a cardiovascular precursor cell [43]. Also, contracting CM has been generated from END2 mouse endoderm-like cells, embryoid bodies, and monolayer cultures [39, 44, 45, 46]. Clinically, safety concerns regarding the transplanted iPSC-cells owing to immunogenic reactions are challenging [47, 48]. Interestingly, emerging protocols using modifying messenger ribonucleic acids (mRNAs) [49] and microRNAs (miRs) to control such adverse effects are promising [50, 51].

The transgenes have been introduced into the host genome via viral integration using retroviruses or lentiviruses [52]. However, this method offers the challenge of reactivating or inactivating host genes, such as *c-Myc*, thus leading to increased tumorigenicity [52, 53]. A study performed on mice and human fibroblasts examined the potential of a family of proteins including Oct4, Sox-2, Klf4, and c-Myc for inducing pluripotency using pMXs-based retroviral vectors and Plat-E cells [53]. The results showed a significant reduction in tumorgenicity without the transduction of Myc retrovirus [53, 54]. In contrast, another study eliminated the introduction of c-Myc to induce iPSCs using Oct4, Sox-2, and Klf4 [54]. These results showed that c-Myc plays a large role in the efficiency and enhanced proliferation of iPSCs [54].

The major limitation of creating iPSCs is to determine the efficiency and the timing. For instance, keratinocytes have proven to be both faster and more efficient compared to fibroblasts [55]. iPSCs have also been created through exfoliated human renal epithelial cells excreted in urine, and isolation of cells from human urine is cost-effective, simple, and easy [56]. Also, the protocol to produce urinary iPSCs (UiPSCs), is faster with a culture time of 2 weeks followed by reprogramming for 3–4 weeks [56]. The resulting UiPSCs exhibited strong differentiation potential to generate all three germ layers *in vivo* and *in vitro * [57].

While efficiency and rate of production of iPSCs is partially determined by the cell source, the iPSCs possibly retain cell-of-origin epigenetic memory [57]. Nonetheless, the usage of iPSCs provides an innovative and exciting platform to engineer CM for translational cardiology.

## 6. Gene Manipulations

**Fig. 1.**
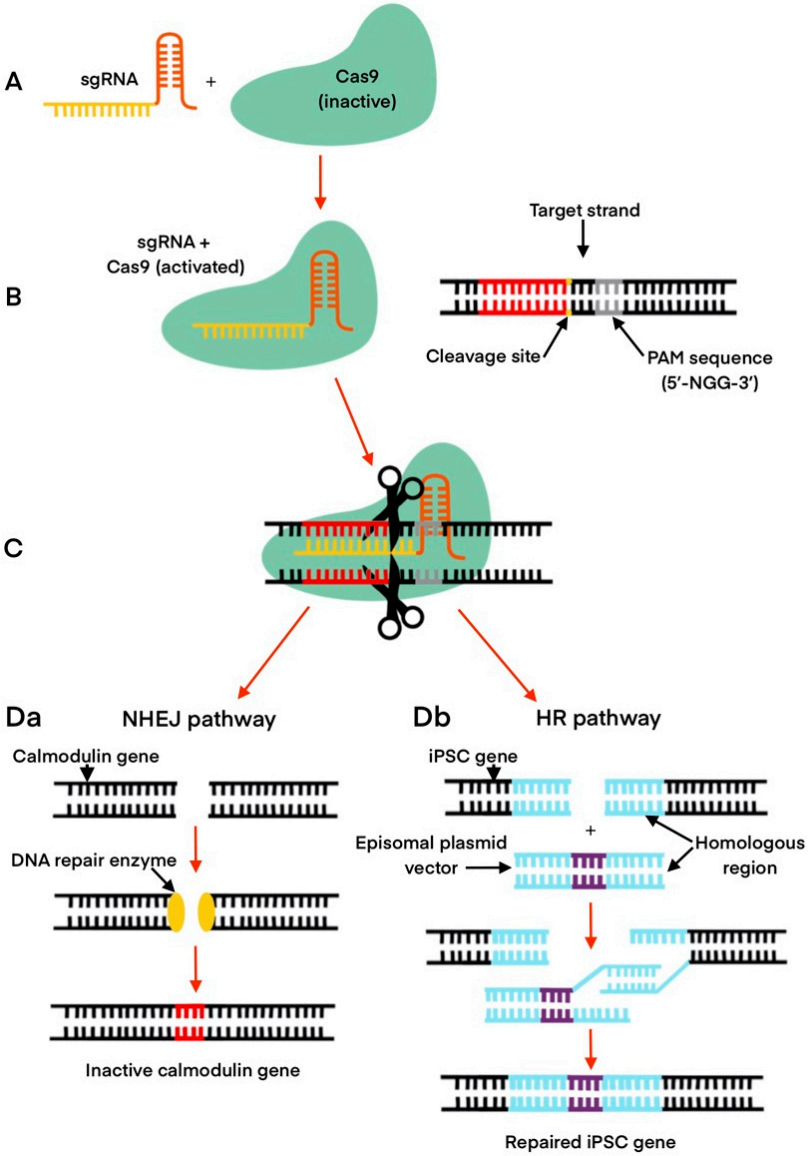
**CRISPR/Cas9 pathways involved in the generation of cardiac iPSCs**. (A) Components required: engineered single guide RNA (sgRNA) and inactivated Cas9 protein. (B) Introduction of sgRNA activates Cas9 to the target region for cleavage. (C) Cas9 makes double strand break three base pairs up from protospacer adjacent motif (PAM). Da, non-homologous end-joining (NHEJ) causes silencing of gene (example: modification of calmodulin gene for treatment of long QT syndrome). Db, homologous repair (HR) repair pathway with the addition of exogenous DNA resulting in edited gene (example: episomal plasmid vector resulting in repaired iPSC gene) [47, 48, 52].

5a. CRISPR: Genetic manipulations have been used to induce pluripotent stem cells where clustered regularly interspaced short palindromic repeats (CRISPR/Cas9) technology is promising in editing the genes for generating iPSCs [58]. In cardiovascular pathology, CRISPR has been successfully employed to alter the genetic make-up of fibroblasts to ensure the regenerative responses [59]. Using fibroblasts or somatic stem cells, an *ex vivo* approach to edit the genes from patient derived cells has proven to be translationally relevant [58]. Similar studies have been shown to modulate immune cells, lipid metabolism pathways, and viral genomes [58]. In a seminal study, human iPSCs were used to study mutant genes in relation to long QT syndrome [59, 60, 61, 62] and using CRISPR technology the mutant allele has been silenced while preserving the normal allele and cell function [60, 61, 62]. Furthermore, studies have shown that CRISPR technology has successfully restored function of human cells in mouse models of Duchenne Muscular Dystrophy (DMD) by editing the exon 44 deletion from cardiac myocytes [63]. Furthermore, ~90% restoration of dystrophin protein in all muscle cells has been achieved in mouse model which is promising as only 15–30% restoration is necessary to provide therapeutic levels to patients [63]. In another seminal study using mice model demonstrated the restoration of the mutated PRKAG2 gene, the key mutation leading to familial WPW (Wolf-Parkinson’s White) syndrome using a combinatorial approach of viral vector with CRISPR/Cas9 to restore of the cardiomyocyte morphology [60]. Translationally, CRISPR targets custom sequences within the genome either directly or indirectly using various sized single guide RNAs (sgRNA) in conjunction with Cas9 systems for site-specific molecules to target mutations or genetic sequences for therapeutic applications [64]. However, off-target effects and ethical considerations are challenging [65]. Overall, CRISPR technology has been widely applied in the studies regarding coronary heart disease, hypertrophic cardiomyopathy, Wolf-Parkinson White Syndrome and calmodulinopathic Long-QT syndrome [59, 60, 61, 62]. The general approach of CRISPR technology in generating cardiac iPSCs is shown in Fig. 1 (Ref. [47, 48, 52]).

5b. ZFN and TALENs: As an extension of CRISPR technology, genetic editing tools such as zinc-finger nucleases (ZFN) and transcriptional activator like effector nucleases (TALENs) are other ways to modify genes within cells. ZFN cleaves DNA at certain sites, thus allowing for the addition or deletion of DNA sequences [61, 66, 67]. The ZFN encompasses two domains: a DNA binding domain and DNA cleaving domain [61, 66, 67].

Once the domain binds its target, a DNA double strand break is induced which is repaired through homologous and nonhomologous end joining [61, 66, 67]. A classic example of this method is evident in HIV and the CCR5 gene on immune cells [66]. Also, the gene encoding the CCR5 receptor in T cells has been knocked out using ZFN technology leading to decreased susceptibly to the HIV virus in the patient [66]. Logically, the DNA sequences within the target cells can be manipulated by ZFN for differentiation towards cardiac lineage or modulating the cell cycle [66]. TALENs were identified from Xanthomonas genus [68, 69] as transcription factors binding to DNA for activating transcription [55, 56]. Christian *et al*. [68] demonstrated that TALENs possess extreme translational potential where highly specific sequencing nucleases are needed to target arbitrary genes within the genome [55, 56]. Hence, the identification of highly specific and custom-tailored molecules are possible for the transcriptional activation of genes allowing the cell programming [68, 69]. For example, the phospholamban gene (*PLN*) codes for a protein that functions to regulate the influx of calcium into cardiomyocytes [69]. Mutations in the *PLN* gene have been implicated in various cardiomyopathies resulting in impaired calcium kinetics and contractility where TALEN correction has successfully restored the calcium homeostasis and contractility [69].

5c. RNA interference: RNA interference (RNAi) has been widely used to inhibit expression of genes by complexing with mRNA molecules preventing translation [70, 71]. MicroRNA (miRNA) and the synthetic form siRNA are routinely used in cardiac research [71, 72]. Interestingly, the inhibition of lipoprotein receptor-related protein 6 (LRP6) via miRNA-LRP6 resulted in increased proliferation of CM along with stem cell differentiation towards CM [72]. Additionally, the deficiency of LRP6 improved heart function with a concomitant reduction in infarct size as evident from MI-mouse model [72]. Furthermore, lipid nanoformulations have been devised to deliver these non-coding RNAs *in vivo*. This process was first approved in 2018 for an anti-transthyretin siRNA for treatment of amyloidosis [71]. Transthyretin mutations result in amyloidosis, hence siRNA targeting the synthesis of this protein can lower the rate of its production [73]. In another study, PCSK9 was targeted in the hopes of modulating LDL levels in healthy individuals and targeting PCSK9 lowered LDL levels in non-human and human primates [73]. The advantage of RNAi is that the molecules formed can be specific and tailored to each subject by measuring the relative expression of targeted molecules in various tissues and the application of PCR techniques for industrial level production. Lastly, a library of sequences is possible allowing the discovery of novel miRNAs, siRNAs and other non-coding RNAs for cardiac applications [74]. However, the challenges including the design of the drugs and the delivery system, lack of clinical translation research, patient selection, and regulatory issues warrant further attention [73]. Overall, multiple studies have proven the impact of RNA interference to downregulate proteins preventing cardiac damage and accelerating cardiac regeneration.

5d. Viral vectors: Forced expression of key transcription factors such as Gata4 (G), Hand2 (H), Mef2c (M) and Tbx5 (T) in fibroblasts resulted in CM-like phenotypes favoring cardiogenic regeneration [75]. An important study demonstrated that the forced expression of these four transcription factors in fibroblasts resulted in mature contractile fibers, but with minimal sarcomere construction [76]. At least three factors (GMT) are essential to induce sarcomere proteins in most of the cells; however, Hand2 in the context of GMT expression dramatically increased the structure and function within the induced CMs [75]. Another study used retroviral genomes containing 6 core transcription factors (GATA4 (G), HAND2 (H), MEF2C (M), MESP1 (Ms), NKX2-5 (N), and TBX5 (T)) to control cardiac gene expression and differentiation [76]. Also, the murine fibroblasts bearing a specific promotor gene that codes for *MHC-GFP* has been involved in the reprogramming of fibroblasts into functional cardiac cells. Additionally, GMHT expression in non-cardiomyocytes in the heart limited fibrosis and improved overall cardiac function [76].

5e. Electrical stimulation: Various experiments have shown that electrical signals induce structural and functional alterations in stem cells and cardiac cells [77]. Amirabad *et al*. [78] demonstrated that iPSCs generated from the fibroblasts of CVD patients induced the expression of CM biomarkers, such as Troponin I, upon electrical simulation. Another study employed differentiation of iPSCs to cardiac lineage by forming embryoid bodies (EB). The electrical stimulation of EB resulted in an increased expression of cardiac genes such as *ACTC1, TNNT2, MYH7*, and *MYL7*, and upregulated various cardio-specific transcription factors and contractile markers. Interestingly, the beating EBs revealed the ability to exchange calcium ions in response to chromotropes, suggesting that the electrical stimulation is essential to promote cardiac differentiation of iPSCs [79].

5f. Hypoxia and ischemia: Hypoxia and ischemia have been reported to be strong triggers for stem cell activation and differentiation [80]. A seminal study showed that in newborn mice, 6 hours of hypoxic insults in cardiac fibroblasts resulted in the reprogramming of cardiomyocyte-like cells, as evident from the elevated levels of cardiac related genes and transcription factors [81]. Also, the secretome derived from human amniotic fluid stem cells (AFSC-S) under hypoxia resulted in the generation of human adult cardiomyocytes suggesting their regenerative potential [82].

## 7. Translational Outcomes

**Fig. 2.**
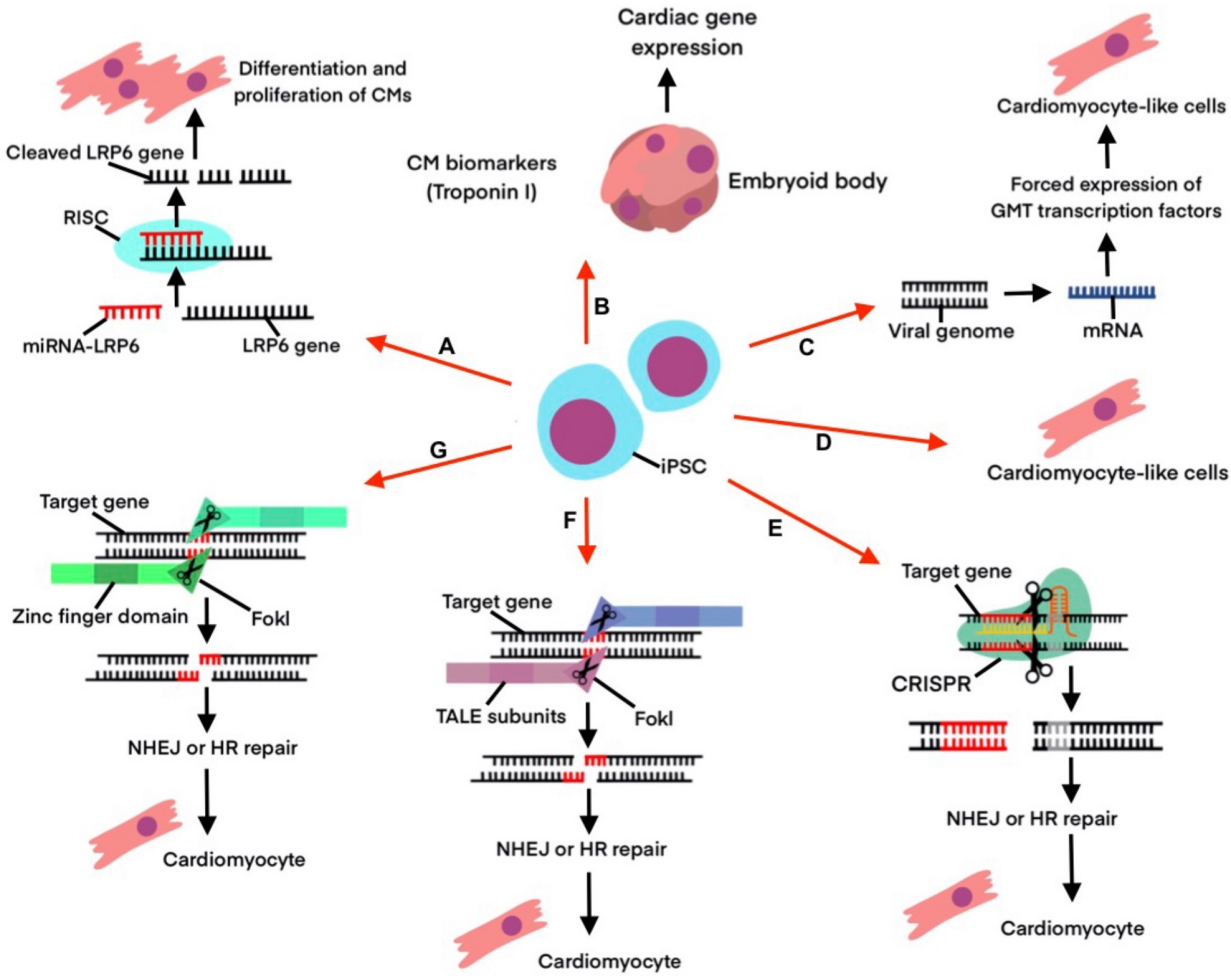
**Pathways for iPSC differentiation into CMs and CM-like cells**. (A) Inhibition of LRP6 via RNAi [72]. (B) Electrical stimulation of iPSCs [78]. (C) Viral genome vectors influencing gene expression [75, 76]. (D) Hypoxia inducing differentiation into CM-like cells [79]. (E) Targeted gene editing via CRISPR [58]. (F) Targeted gene editing via TALENs [69]. (G) Targeted gene editing via ZFN [66].

Interestingly, the above-mentioned strategies (Fig. 2, Ref. [58, 66, 69, 72, 75, 76, 78, 79]) are already being explored in human clinical trials. The Stem Cell Infusion in Patients with Ischemic Cardiomyopathy (SCIPIO) Phase I Trial was the first-in-human use of autologous c-kit+ cardiac stem cells (CSCs) where HF patients with ischemic etiology demonstrated a pronounced increase in left ventricular ejection fraction (LVEF) and regional EF within the CSC-infused territory [83]. About 22.7% decrease in infarct size was observed 4 months following post-CSC infusion and a 30.2% decrease at 12 months post-infusion was reported alongside salient improvement in cardiac regeneration as evident from cardiac magnetic resonance (CMR) where patients presented viable tissue growth even after 1 year follow-up [83]. Overall, SCIPIO underscored a potential new treatment for patients with severe HF and ischemic cardiomyopathy and was the first study to demonstrate the ability of these CSCs to be extracted in the operating room during a CABG surgery [83]. Clinically, the ${\mathrm{C-kit}}^{+}$ cardiac stem cells have been meticulously studied in both basic and clinical investigations for cardiac cell regeneration with controversial findings [84, 85, 86]. It was originally thought to be a primary driver for post-MI myocardium regeneration [84, 87, 88] and the expression of c-kit within cardiac cells have been assumed as an identification of a CSC [86], despite its expression by a diverse cardiac cell population [85, 89]. Disregarding the heterogeneity, the c-kit has led to the dispute in its role in determining CSC fate and its expression alone is not a predictor of CSCs [86, 90]. A seminal study found that although endogenous c-kit+ cells produce new cardiomyocytes, albeit at an insignificant level [91]. Another study concluded that c-kit+ cells are endothelial cells but not CSCs based on the expression status and co-localization with other cardiac progenitor markers such as cardiac troponin T [92]. However, new studies investigated c-kit+ cells expression with debatable results and contradictory conclusions stemming from the advantages and disadvantages of different tools and methods utilized in isolating the c-kit locus [90]. Thus, a more precise tracing tool could help elucidating the role of c-kit in CSCs warranting further detailed investigations.

Importantly, the bone marrow derived mononuclear cell (BMMNCs) exhibited successful clinical trials as evident from the improved contractility and perfusion post-transplantation. Interestingly, the patients without the cell transplant remained unaltered on considering neovascularization, myocardium regeneration [93], and infarction wall movement [93] with a concomitant decrease in LVESVI and LVEDVI [94]. Skeletal myoblast transplantation has also been studied in patients with MI. In a one-man study, the patient received 33 cell suspensions originating from the patient’s vastus lateralis muscle into the area of infarction on the posterior wall of his left ventricle. They displayed improved clinical status with an increase in LVEF and improvement in posterior wall contraction [95]. This study substantiates the potential usage of skeletal myoblast cells for cardiac tissue, opening novel avenues in translational cardiology.

In the Cardiosphere-Derived autologous stem cells to reverse ventricular dysfunction (CADUCEUS) trial, the patients with history of recent MI received Cardiosphere-derived cells (CDCs), resulting in viable heart mass, regional contractility, and thickening of the regional systolic wall [96]. The CDCs were grown from autologous endomyocardial biopsies taken from patients with MI within 30 days. This trial demonstrated proof-of-concept for the clinical study of CDCs and serves as early evidence for regeneration therapy. Bone marrow derived stem cells (BMSCs), bone marrow hematopoietic stem cells (BMHSCs), bone marrow derived endothelial cells (BMEPCs), adipose derived cells, cardiac stem cells, embryonic stem cells, and other cell types have shown promise translationally into clinical trials [97]. An important study revealed that murine models injected with BMSCs into the myocardium resulted in improved cardiac function demonstrating the therapeutic benefit [98]. In MI patients, injection of BMHSCs and BMEPCs at the infarcted zone improved left ventricular ejection fraction (LVEF) and myocardial tissue perfusion [99, 100].

Despite these astounding advances made in the last decade, the efficacy of translational research remains a challenge, as many of the preclinical studies lack the rigor needed to effectively translate to real patients [101]. The Transnational Alliance for Regenerative Therapies in Cardiovascular Syndromes (TACTICS) proposed an improvement in quality of preclinical research alongside better communication and collaborative efforts to meet translatability and to improve quality of life of ischemic patients [101]. The seminal translational findings are displayed in Table 2 (Ref. [83, 93, 95, 96, 98, 99, 100, 102, 103, 104]).

**Table 2.** **Summary of the translational therapeutic agents, their approach and outcomes**.

Therapeutic agent	Approach	Outcome	Reference
Cardiac stem cells	Infusion of autologous c-kit+ cardiac stem cells extracted during CABG	Increased left ventricular ejection fraction and regional ejection fraction, decreased infarct size, and viable tissue growth	[83]
Bone marrow derived mononuclear cells	Cell transplant of autologous mononuclear bone marrow cells into the artery supplying the infarcted area	Neovascularization and myocardium regeneration, resulting in improved contractility and perfusion	[93]
Skeletal myoblasts	Injection of skeletal myoblasts into area of infarct in patients with previous myocardial infarction and/or heart failure	Increased left ventricular ejection fraction and improved contraction in infarcted area, as well as improved symptoms	[95, 102, 103, 104]
Autologous stem cells	Patients with history of myocardial infarction <30 days prior were assigned randomly to control group or to receive cardiosphere-derived cells grown from endomyocardial biopsy	Increased viable heart mass, regional contractility, and thickening of regional systolic wall	[96, 98]
Bone marrow derived stem cells	Injection of CD133+ cells into the myocardium or recruitment via cytokines	Improved cardiac function via production of new cardiomyocytes and coronary blood vessels	[98, 99, 100]
	Also, in conjunction with CABG		

## 8. Summary 

The programmed cell types destined for replenishing the CM in the surviving myocardium offer promising translational opportunities in the management of HF. The solitary use or combination of genetic manipulations and exogenous stressors are being explored within cardiac stem cells to make strides towards possible therapeutic ends of cardiac pathology. There are numerous cell types and molecular mediators involved in cardiac tissue repair; proper understanding of the underlying molecular signaling is required for cell programming techniques to be harnessed and exploited for cardiac regenerative strategies. Stem cells as a potential treatment have been used in multiple approaches in current research, including cutting edge CRISPR technology gearing towards regenerative cardiology. Findings from animal models and human trials have shown progress unveiling the immense promise for cell reprogramming; however, further in-depth investigations are warranted to address the existing challenges in the application of programmed cells for cardiac regeneration. Even so, the programmed/engineered cells offer strong translational potential as future therapeutics for the accelerated regeneration/healing of the failing myocardium.
